# Physiologically based pharmacokinetic modeling of tafenoquine and evaluation of transporter-mediated drug–drug interactions

**DOI:** 10.3389/fphar.2026.1880776

**Published:** 2026-07-17

**Authors:** Luisa Oliveira Santos, Pieter Annaert, Francine Johansson Azeredo

**Affiliations:** 1 Laboratory of Pharmacokinetics and Pharmacometrics, Faculty of Pharmacy, Federal University of Bahia, Salvador, Brazil; 2 Drug Delivery and Disposition, Department of Pharmaceutical and Pharmacological Sciences, KU Leuven, Leuven, Belgium; 3 BioNotus, Niel, Belgium; 4 Center for Pharmacometrics & System Pharmacology, Department of Pharmaceutics, College of Pharmacy, University of Florida, Gainesville, FL, United States

**Keywords:** drug-drug interactions (DDI), malaria, pharmacometrics, physiologically based pharmacokinetic (PBPK) modeling, tafenoquine

## Abstract

**Introduction:**

The aim of the study was to develop a physiologically based pharmacokinetic (PBPK) model of tafenoquine (TQ), a new drug for the radical cure of *Plasmodium vivax* malaria and assess drug-drug interactions with substrates of the transporters OCT2 and MATE1.

**Methods:**

A whole-body PBPK model was built using PK-Sim® to simulate 100 adults aged 19–53 years weighing 51–108 kg. Hepatic clearance was incorporated into the model. Literature data on the oral administration of TQ were used to verify the model. The qualified model was used to simulate co-administration with metformin (MTF), an OCT2 and MATE1 substrate, and assess drug-drug interactions.

**Results and Discussion:**

The PBPK model presented a good descriptive and predictive performance for plasma concentration-time profiles. Quantitative measures of the model performance fell within the 2-fold acceptance criteria when predictions were compared to observed values. Co-administration of TQ with MTF presented no alteration of MTF’s exposure. Model simulations suggest that there is no significant interaction between TQ and substrates of OCT2/MATE1, such as MTF.

## 1 Introduction

Tafenoquine (Krintafel®) is a new 8-aminoquinoline drug for the radical cure of *Plasmodium vivax* malaria and malaria prophylaxis. It is not first-line therapy, as primaquine is well established. However, tafenoquine (TQ) is a single-dose alternative that supports treatment compliance compared to longer treatment regimens (Pereira et al., 2011; Frampton, 2018). Since malaria is an infectious disease with a global burden that is still rising, it is essential to investigate new strategies for malaria elimination (World Health Organization, 2024a). There were 263 million cases in 2023, primarily related to *Plasmodium falciparum*. However, *P. vivax* malaria is also experiencing a growing number of cases, like the 2014 scenario (World Health Organization, 2024a). Epidemiological trends support the rationale for new treatment strategies.

According to the Biopharmaceutics Classification System (BCS), TQ is a Class II drug with low solubility and high permeability, and exhibits pH-dependent solubility (U.S. Food and Drug Administration, 2025). TQ has a long half-life of approximately 14 days in humans, exhibits high protein binding (>99.5%) in humans and animals, and tissue recovery studies suggest possible lysosomal trapping, as with other basic drugs. Additionally, it is subject to Cytochrome P450 (CYP2D6) mediated metabolism (U.S. Food and Drug Administration, 2025; Assmus, Houston, and Galetin, 2017). The major metabolite of TQ is 5,6-ortho-quinone tafenoquine as a product of a hydrolysis reaction, other metabolic pathways seem to be part of TQ metabolism in humans too (Ebstie et al., 2016). TQ absorption, distribution, metabolism, and excretion (ADME) are not yet well characterized. Barber et al. (2023) showed that the plasma concentration-time profile of TQ in humans exhibits a second peak after 7–10 days of administration. This second peak behavior can be explained by enterohepatic circulation (EHC), lysosomal trapping affecting drug distribution, and a longer half-life affecting the concentration-time profile due to insufficient data collection (Lehr et al., 2009; Metsugi et al., 2008; Suttle, Pollack, and Brouwer, 1992). It is important to better understand TQ pharmacokinetics (PK) to ensure safe therapy.

Regarding safety in larger populations, TQ carries label warnings about its potential risk of drug-drug interactions (DDIs) with substrates of the organic cation transporter 2 (OCT2) and multidrug and toxic compound extrusion transporters (MATEs) (U.S. Food and Drug Administration, 2025; GlaxoSmithKline, 2018). There is no evidence of TQ undergoing or causing a relevant CYP-mediated DDI (U.S. Food and Drug Administration, 2025). Hence, there remains a knowledge gap in the ADME profile of TQ, along with limited clinical data, providing an opportunity to use tools such as physiologically based pharmacokinetic (PBPK) modeling and simulation. PBPK models provide a framework for integrating information from multiple sources, including physiology, population characteristics, and drug properties. This allows the modeler to extract the maximum insight from the available data and gain a mechanistic understanding of the processes underlying a drug’s PK (U.S. Food and Drug Administration, 2018; European Medicines Agency, 2018; Jones and Rowland-Yeo, 2013). Modeling and simulation (M&S) tools are essential for drug development and enable improving rationality and safety in pharmacotherapy (Usman et al., 2023). Thus, the aims of this work were to develop a PBPK model for TQ and to assess the transporter-mediated DDI highlighted in the label.

## 2 Materials and methods

### 2.1 Software

The PBPK model was developed using PK-Sim® version 11.3 as part of the open-source modeling software, the Open Systems Pharmacology Suite (Bayer Technology Services, https://www.open-systems-pharmacology.org/). All published study data were digitized using WebPlotDigitizer (version 5.2, Ankit Rohatgi, Pacifica, CA, United States). Model input parameter optimization and sensitivity analysis were performed in PK-Sim®. Microsoft Excel 2016 version 16.0 (Microsoft Corporation, Redmond, WA, United States) and R Studio version 4.1.748 (RStudio Incorporation, Boston, MA, United States) running R version 4.4.0 (R Foundation for Statistical Computing, Vienna, Austria, 2020) were used for model performance, statistical calculations, and plot generation.

### 2.2 PBPK model building

Model building was performed using a stepwise procedure. It began with an intensive literature search to gather physicochemical data and quantitative information on ADME mechanisms, along with published clinical studies. The gathered data were used for model development, along with TQ peroral (po) plasma profiles. Data were split into a training dataset (20%), used for model building and parameter optimization, and a test dataset (80%) for model evaluation. All selected studies were human studies; four used a single dose of TQ tablet or capsule, covering a dose range of 200–600 mg, and one used a prophylactic TQ dose of 200 mg. Malaria prophylaxis with TQ is a long-term treatment. It includes a loading regimen for 3 days, a maintenance regimen after 7 days of the last loading dose, and a final prophylactic regimen after the previous maintenance dose. The Supplementary Material summarizes each study and the allocation to the training or the test dataset.

A whole-body PBPK model was built using PK-Sim® to simulate 100 adults aged 19–53 (50% female) weighing 51–108 kg. The population was used for model development and evaluation. Physiological and compound-relevant parameters from the literature were used during model development. Those not found in the literature were estimated and adjusted using parameter optimization with the Levenberg-Marquardt algorithm, with 10 runs performed using multiple starting values in PK-Sim®. The specific intestinal permeability was one of the optimized parameters, along with others related to drug disposition. Some of the data used for model building showed a second peak in the plasma concentration-time profiles (Barber et al., 2023). Different approaches were tested to explain TQ’s distribution and this second peak. All calculation methods for partition coefficients and cellular permeability were tested, and a Weibull function was used to describe the dissolution behavior of the dosage forms. After selecting the best calculation methods, TQ’s distribution and elimination still needed adjustment. To improve model description, a lysosomal sequestration mechanism was incorporated, along with an intracellular binding partner, in the main organs, such as the kidney and lungs. No data on TQ in human tissues were available to validate this idea, and the available mouse data presented important limitations for establishing a PBPK model in mice. So, we rejected it. Another alternative for adjusting TQ’s profiles is related to its biliary clearance and the enterohepatic recirculation process described by Brueckner et al. (1998). A fraction of the biliary secreted compound continuously entering the duodenum was incorporated into the population, and a biliary clearance of the compound. In the lack of literature values, biliary clearance was optimized to fit the observed data. However, this approach implies a dominant role for biliary clearance in tafenoquine elimination, which is not supported by the available literature. In addition, the timing of the second concentration peak is inconsistent with typical enterohepatic recirculation processes, as it occurs substantially later than expected for biliary recycling-driven re-entry. Together, these observations led to the rejection of enterohepatic circulation as a plausible structural mechanism. The final model, therefore, incorporated a hepatic metabolic clearance pathway, combined with a Weibull function, to describe the prolonged distributional and terminal-phase characteristics of tafenoquine. This structure provided the most parsimonious and stable description of the observed data while avoiding unsupported mechanistic assumptions regarding biliary recycling. Sensitivity analyses were performed to evaluate the influence of changes in input parameters on the final PBPK model, especially those that had been optimized or had a substantial impact on the model due to PK-Sim®'s calculation methods. Also, hepatic impairment was assessed considering hepatic elimination. It was implemented with varying conditions based on the Child-Pugh classification, as described by Edginton and Willmann (2008). Simulations were built with the TQ clinical and prophylactic doses.

### 2.3 PBPK model evaluation

To assess model performance, multiple methods were used: visual comparison between predicted population plasma profiles and observed data; plotting predicted plasma concentrations against the corresponding observed values on predictive performance plots; comparison of predicted maximum concentration (Cmax) and the predicted area under the systemic drug concentration-time curve from time zero to the time of the last concentration (AUClast) with their respective literature values (U.S. Food and Drugs Administration, 2018; European Medicines Agency, 2018; Kuemmel et al., 2020). The quantitative measures of model performance were the mean relative deviation (MRD) of all predicted plasma concentrations (Equation 1) and geometric mean fold error (GMFE) of all predicted AUClast and Cmax values (Equation 2). These quantitative measures indicate adequate model performance when falling within a 2-fold predictive error (Maharaj et al., 2020).

$$MRD=10^{X};x=\sqrt{\frac{\sum_{i=1}^{k}\left(\log_{10}Cpred,i-\log_{10}Cobs,i\right)}{k}}$$ (1)

where *Cpred,i* is the predicted plasma concentration; *Cobs,i* is the corresponding observed plasma concentration; and k is the sample size.

$$GMFE=10^{X};x=\frac{\sum_{i=1}^{m}\left|\log_{10}\left(\frac{pred\;PK\;parameter,i}{obs\;PK\;parameter,i}\right)\right|}{m}$$ (2)

where pred PK parameter,i is the predicted AUClast or Cmax value; obs PK parameter, i is the respective observed AUClast or Cmax value, and m is the number of studies.

### 2.4 Drug-drug interaction simulation

TQ PBPK model was combined with the metformin (MTF) PBPK model developed by Hanke et al., 2020 to assess the DDI between TQ and MTF. The US Food and Drug Administration recommends MTF as an OCT2/MATE1 victim drug for clinical DDI studies (U.S. Food and Drug Administration, 2019). There is no published clinical data on this DDI between TQ and MTF, so the interaction was created *in silico* to evaluate its impact on MTF PK profile. The predictive performance of PBPK modeling for transporter-based DDIs has not been established yet due to knowledge gaps in transporter biology and kinetics (Wagner et al., 2015a; Wagner et al., 2015b). Therefore, for transporter inhibitors, it is recommended to establish and verify models for both the perpetrator and the transporter substrate (Taskar et al., 2020; Snoeys et al., 2016). The MTF model was re-verified using the same metrics as for TQ. The results are presented in the Supplementary Material. The interaction type is not established, and TQ is not reported as a transporter substrate in the literature. Different inhibition mechanisms were tested. Due to a lack of literature data to determine the inhibition mechanism of TQ on MTF, a conservative approach was adopted, assuming competitive inhibition. Varma et al. (2012) also describe competitive inhibition using an *in vitro* Ki value. The TQ inhibition constants (Ki) for OCT2 and MATE1 were extracted from the literature and incorporated into the perpetrator model: OCT2 Ki = 0.28 μmol/L and MATE1 Ki = 1.80 μmol/L. These values are available at the FDA multidisciplinary review of the drug. They are based on an *in vitro* study using human embryonic kidney 293 (HEK293) cells expressing the transporters (U.S. Food and Drug Administration, 2025). To evaluate the impact of concomitant use of MTF and TQ, multiple dosing simulations were performed using regular doses of MTF (500 mg once daily and twice daily, 850 mg once daily), the recommended dose of TQ for *P. vivax* malaria (300 mg), and the prophylactic dose (200 mg) (American Diabetes Association, 2025; U.S. Centers for Disease Control and Prevention, 2024; World Health Organization, 2024b). Higher TQ dose regimens were used to assess TQ DDI with these transporters. Clinically relevant dose regimens of MTF were selected for the negative control (NC) of the interaction. Simulations with non-functional transporters (Vmax = 0) were used as a positive control (PC). Since there are no published data on this interaction, the observed data used were from studies with MTF alone. The performance of the DDI model was evaluated by comparing predicted versus observed victim drug plasma concentration-time profiles when administered alone and during co-administration, as well as comparing the ratios of AUClast (Equation 3) and Cmax (Equation 4) for the administration of MTF alone and with TQ. Also, a fold error (FE) assessment (Equation 5) was performed with the same parameters above to check the impact of this potential DDI.

$$DDI\;AUClast\;ratio=\frac{AUClast\;victim\;drug\;during\;co-administration}{AUClast\;victim\;drug\;alone}$$ (3)

$$DDI\;Cmax\;ratio=\frac{Cmax\;victim\;drug\;during\;co-administration}{Cmax\;victim\;drug\;alone}$$ (4)

$$FE=\frac{predicted\;PK\;parameter}{observed\;PK\;parameter}$$ (5)

## 3 Results

### 3.1 PBPK model building and evaluation

**FIGURE 1:**
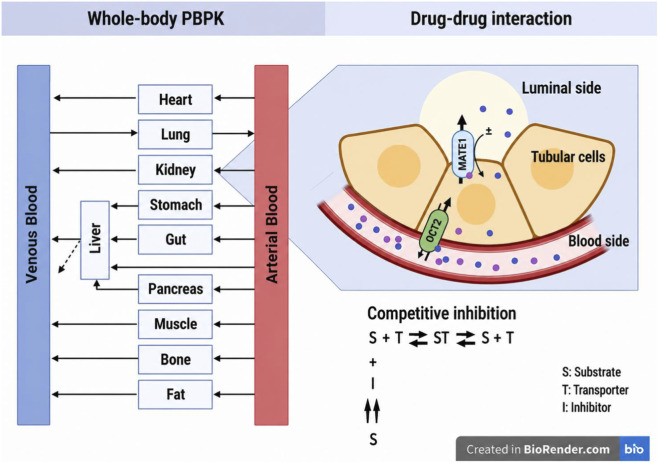
Schematic workflow of the physiologically based pharmacokinetic (PBPK) model and its drug-drug interaction application. The whole-body PBPK model (left) represents drug distribution across major organs and tissues; Gut is gastrointestinal tract; and the dashed arrows represent elimination. The drug-drug interaction (DDI) scheme (right) represents the transporters’ location in kidney tubular cells - OCT2 is basolateral and MATE1 is apical - and the potential interaction between tafenoquine (purple dots) and metformin (blue dots), a competitive inhibition was created.

**FIGURE 2:**
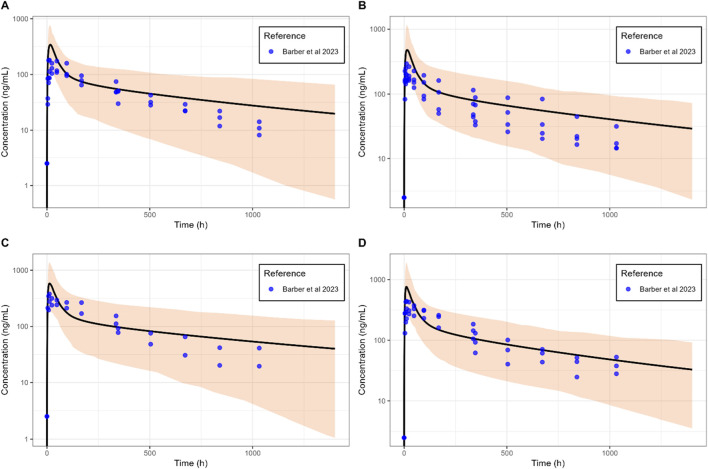
TQ plasma concentration-time profiles (semilogarithmic) of the training dataset. Population predictions plasma concentration-time profiles of TQ 200 mg **(A)**, 300 mg **(B)**, 400 mg **(C)**, and 600 mg **(D)** single dose. Observed data are shown as dots, the population predicted arithmetic mean is shown as lines, and the shaded area represents the 5th and 95th percentiles of simulations.

**FIGURE 3:**
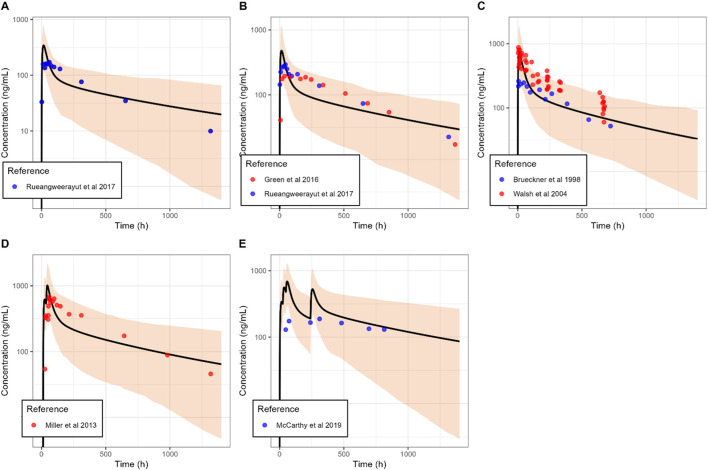
TQ plasma concentration-time profiles (semilogarithmic) of the test dataset. Population predictions plasma concentration-time profiles of TQ 200 mg **(A)**, 300 mg **(B)**, 600 mg **(C)** oral single dose; 450 mg once-daily for 2 days **(D)**; and prophylactic dose **(E)**. Observed data are shown as dots, the population predicted arithmetic mean is shown as lines, and the shaded area represents the 5th and 95th percentiles of simulations.

**FIGURE 4:**
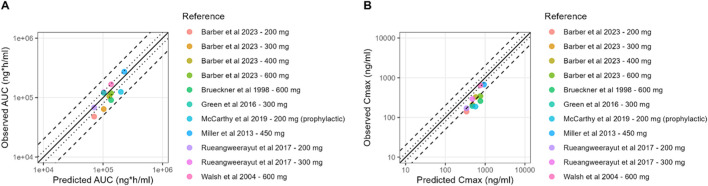
Predictive performance plots for the PK parameters. Predicted versus observed AUClast values **(A)** and predicted versus observed Cmax values **(B)**. Observed data are shown as dots, the black solid line represents the line of identity (perfect prediction), dotted lines represent a 1.25-fold deviation, and dashed lines represent a 2-fold deviation.

**FIGURE 5:**
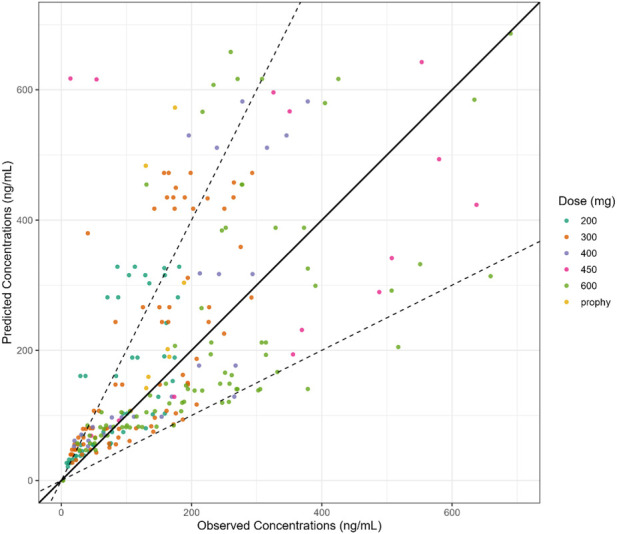
Predictive performance plot for the PBPK model. Observed concentrations are shown as dots colored by dose, the black solid line represents the line of identity (perfect prediction), and the dashed lines indicate a 2.0-fold error range.

**FIGURE 6:**
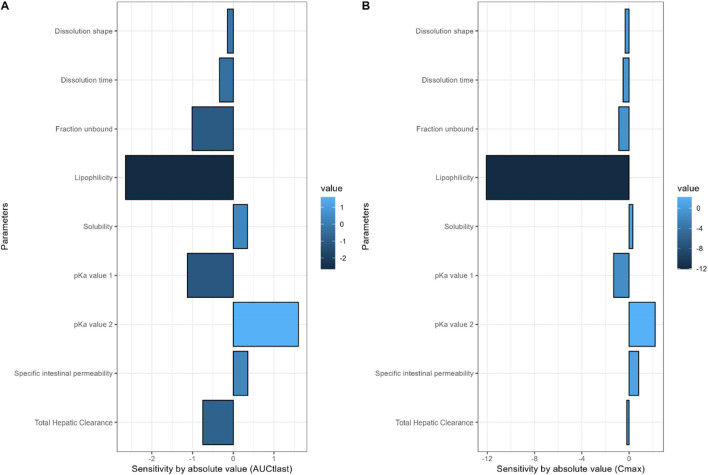
Sensitivity analysis of the TQ PBPK model. Sensitivity to single parameters measured as a change of the AUClast [ng*h/mL] **(A)** and the Cmax [ng/mL] **(B)** from the 300 mg single oral dose.

A whole-body PBPK model of TQ was developed and evaluated using four clinical studies of single-dose oral TQ administration with different dose regimens (200–600 mg) and one study with a prophylactic dose of TQ for travelers. The overall modeling workflow and DDI application are detailed in Figure 1. A specific hepatic clearance was implemented in the model to describe TQ elimination. The value was based on an *in vitro* microsomes assay, but it was optimized to capture the observed data. The Weibull function has two optimized parameters that help describe the observed data: dissolution time and shape. Plasma concentration-time profiles of 300 mg without the Weibull function had higher exposure (102,713.00 ng*h/mL vs. 140,979.80 ng*h/mL) and Cmax (478.22 ng/mL vs. 771.50 ng/mL). The final parameters used in the human PBPK model are shown in Table 1. The final PBPK model accurately describes the observed TQ plasma concentrations in healthy volunteers, as demonstrated in Figures 2, 3. Predicted plasma profiles were in concordance with observed data. Figure 4 shows the predictive performance plots comparing predicted vs. observed AUClast and Cmax values of all studies used. Predicted AUClast and Cmax values fell within the 2-fold acceptance criteria. FE values for AUClast are between 0.81 and 1.60, and for Cmax, between 1.18 and 3.04, with an overall GMFE mean equal to 1.19 and 2.00, respectively (Supplementary Table S2). The mean MRD was 1.23, ranging from 1.07 to 1.72 (Supplementary Table S2). The overall metrics fulfilled the acceptance criteria. The plasma concentration predictive performance plot comparing predicted vs. observed values is in Figure 5, where most observed concentrations lie within the 2-fold acceptance criteria, indicating that the model provides an overall adequate predictive performance across doses. Even though some points are not captured by the current model. Sensitivity analysis for AUClast and Cmax is presented in Figure 6. Hepatic impairment scenarios were simulated as Child-Pugh A, B, and C. Overall, they presented a progressive impact on TQ exposure with higher AUC and slower elimination (Supplementary Table S3).

**TABLE 1:** Input parameters for the TQ PBPK model.

Parameter	Unit	Value used	Description	Reference
MW	[g/mol]	463.50	Molecular weight	DrugBank (2025)
pKa 1 (base)	​	8.74	Acid dissociation constant	Charman et al. (2020)
pKa 2 (base)	​	6.0	Acid dissociation constant	Charman et al. (2020)
log D 7.4	​	4.24	Lipophilicity	Charman et al. (2020)
Solubility (pH 7)	[mg/L]	1.37	Solubility	DrugBank (2025)
Fu	​	0.002	Fraction unbound in plasma	Charman et al. (2020)
Main binding protein	​	HSA	​	Zsila et al. (2008)
Specific intestinal permeability	[cm/min]	4.72E-4	Intestinal permeability	​
Partition coefficients	​	PK-Sim standard	Calculation method for cell to plasma coefficients	​
Cellular permeability	​	Charge-dependent schmitt	Calculation method for permeation across cell membranes	​
Specific hepatic clearance	[1/min]	5.42^a^	*In vitro* metabolic rate in the presence of liver microsomes	​
Tablet Weibull time	[min]	801.40^a^	Dissolution time (50% dissolved)	​
Tablet Weibull shape	​	0.61^a^	Shape parameter of the weibull function	​
Ki OCT2	[μmol/L]	0.28	Inhibition constant against OCT2	FDA, no date
Ki MATE1	[μmol/L]	1.80	Inhibition constant against MATE1	FDA, no date

^a^ Optimized values.

### 3.2 Drug-drug interaction simulation

**FIGURE 7:**
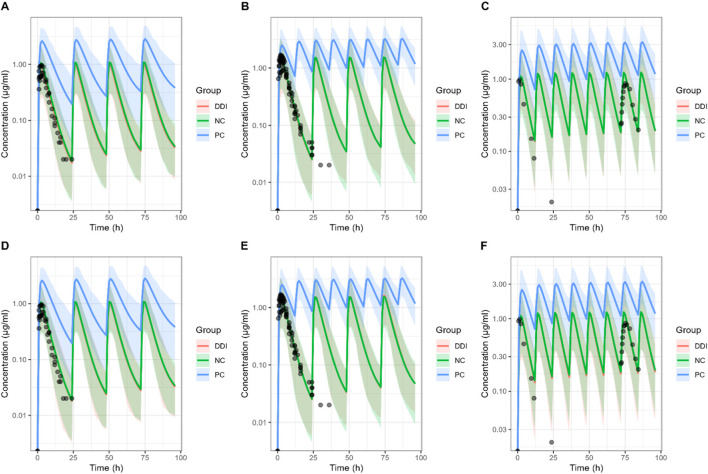
MTF plasma concentration-time profiles (semilogarithmic). Population predictions plasma concentration-time profiles of MTF 500 mg qd with TQ 300 mg **(A)** and with TQ prophy **(D)**; MTF 850 mg qd with TQ 300 mg **(B)** and with TQ prophy **(E)**; MTF 500 mg bid with TQ 300 mg **(C)** and with TQ prophy **(F)**. Observed data are shown as dots, population predicted arithmetic mean is shown as lines, and the shaded area represents the 5th and 95th percentiles of simulations divided by negative control (NC), positive control (PC), and coadministration (DDI).

The results regarding the re-evaluation of the MTF’s previously published data are presented in Supplementary Table S4. The plasma concentration-time profiles of MTF with and without TQ co-administration are shown in Figure 7. Dose regimens for TQ included treatment of *P. vivax* malaria (300 mg single dose) and prophylaxis. Multiple dosing simulations were performed to assess the DDI potential of TQ with higher doses (400 and 600 mg) (Supplementary Figure S1). DDI ratios for AUClast and Cmax of MTF administered alone and with TQ varied between 0.91 and 2.09, with FE lower than 1.30 and 1.42 for AUClast and Cmax, respectively (Supplementary Table S5). This result indicates that TQ is a weak inhibitor according to the FDA and the International Council for Harmonization of Technical Requirements for Pharmaceuticals for Human Use (ICH, 2024) inhibitor classification (U.S. Food and Drug Administration, 2019; ICH, 2024). The potential DDI based on *in vitro* studies does not seem clinically relevant, considering the *in silico* simulations performed.

## 4 Discussion

TQ is a new drug with evidence recommending its use in *P. vivax* malaria treatment and prophylaxis for adults, and it is investigated for other diseases (Hanke et al., 2020; U.S. Centers for Disease Control and Prevention, 2024; World Health Organization, 2024b). This work has established a comprehensive whole-body PBPK model of TQ, integrating the current knowledge of TQ properties. The model was evaluated as a precision tool to assist dosage in different clinical scenarios. This model has limitations due to limited knowledge of TQ properties and ADME processes, particularly absorption and excretion, which were the main parameters optimized. Additionally, there is a lack of intravenous (IV) data on TQ plasma concentration-time profiles and tissue concentrations in humans or animals. IV data could provide a better description of TQ kinetics without interference from an absorption phase, thereby reducing uncertainties (Kuepfer et al., 2016). This lack of knowledge affected assumptions and parameter optimization to describe the observed data.

Some assumptions were made during simulations to compensate for the lack of literature data: uniform drug distribution across tissues and hepatic clearance as the primary route of elimination, with no specific enzymes listed. Animal data indicate high liver accumulation of TQ, with a 70-h half-life in the liver (Li et al., 2014; Pottenger et al., 2024). In the current PBPK model, kidney accumulation (Supplementary Figure S2) increased when the intracellular pH was acidified to mimic a lysosomal environment (Schmitt et al., 2019). These findings, together with the pronounced distribution of TQ, may be related to lysosomal sequestration, a phenomenon described for other aminoquinolines and other basic lipophilic drugs (Schmitt et al., 2019; MacIntyre and Cutler, 1988). Although these data were obtained in mice, due to limitations in establishing a mouse model, it was assumed that the distribution was uniform across tissues. Brueckner et al. (1998) describe biliary excretion with enterohepatic circulation based on data from TQ development. These are unpublished data from the Walter Reed Army Institute of Research and other facilities, which was a limitation in validating these processes. The development of this model highlights the knowledge gap on TQ ADME. The excretion profile reported in rats after a single dose indicates that feces are the major route, followed by bile (U.S. Food and Drug Administration, 2025). In our human PBPK model, feces were also a major route of excretion (Supplementary Figure S3). The model fractions of drug dissolved and absorbed into the intestinal mucosa are around 0.8 and 0.6, respectively, indicating that TQ is primarily eliminated in feces, given the higher fraction of drug dissolved than absorbed (Supplementary Figure S4).

The sensitivity analysis results in Figure 6 show that 9 parameters have a meaningful impact on the model’s performance. They are related to the absorption, distribution, and elimination of TQ. Fraction unbound, lipophilicity, and both pKa values are important to the calculation methods of partition coefficients and cellular permeability. Their impact on the model’s performance highlights the lower permeability of charged species and the pH-dependent distribution, as expected for basic drugs (Schmitt et al., 2019). Specific intestinal permeability and the Weibull function parameters were optimized to fit the observed data. The dissolution profile of BCS Class II drugs is influenced by solubility, formulation, drug nature, and the lumen (Tsume et al., 2014). This optimization allows the model to better describe TQ dissolution and absorption, thereby affecting its exposure. Total hepatic clearance affected TQ exposure. The literature reports its slow metabolization as part of drug elimination. Contradictory evidence regarding TQ’s CYP2D-mediated metabolism in murine and human models underscores the need for new studies on TQ metabolism to better understand which enzymes are responsible (Chu and Hwang, 2021). Hepatic impairment seems to influence TQ accumulation in the body (Supplementary Table S3). Clarifying the elimination pathway of TQ and its key processes is an important step toward optimizing the TQ dose regimen and treating *P. vivax* malaria in special populations.

Figures 2, 3 show the model-prediction curves, with the observed data falling within the 90% confidence interval (CI). In the test dataset, most observed concentration–time data were well captured within the model-predicted confidence intervals, except for Walsh et al. (2004) and McCarthy et al. (2019), where partial deviations were observed. Based on the available demographic information (Supplementary Table S1), one of these studies included a relatively low proportion of male participants, which may have contributed to differences in exposure patterns, although a similar sex distribution was also present in the 300 mg cohort of Barber et al. (2023), where model predictions adequately captured the observed data. For the remaining study, the lack of reported racial background limits interpretation, particularly given the potential for between-study differences in population structure and associated covariates. Importantly, previous clinical pharmacogenetic evaluation of tafenoquine by St Jean et al. (2016), which specifically investigated the impact of CYP2D6 polymorphisms and reduced metabolic capacity, did not identify a clinically meaningful effect on tafenoquine exposure. This supports the assumption that CYP2D6 genetic variability is unlikely to be a primary driver of the observed between-study differences in the current analysis, and is consistent with the model structure, which does not include CYP2D6-mediated clearance as a relevant determinant of tafenoquine disposition. Other than that, the visual analysis indicates good descriptive and predictive behavior in the plasma concentration-time profiles of TQ within a dose range (200–600 mg) that includes both the clinically recommended dose for P. vivax malaria treatment (300 mg) and the prophylactic dose (Figures 4, 5). This behavior is confirmed by the statistical analysis, with MRD and GMFE values meeting the acceptance criteria (Supplementary Table S2). Once again, the results confirm the model’s capability to describe the observed TQ data. However, a few observed data points fall outside the acceptance criteria, and several points deviate from the identity line in Figure 5. The points above and below the identity line, along with the criterion limits, suggest that the model underpredicts or overpredicts concentrations in certain individuals or studies. Such deviations can arise from differences in demographic characteristics across studies or interindividual variability not fully captured by the model (Maharaj et al., 2020; Mould and Upton, 2013; Nguyen et al., 2017). The absence of a clear trend above or below the line indicates remaining unexplained variability and the sources should be explored (Maharaj et al., 2020; Mould and Upton, 2013; Nguyen et al., 2017).

The validated PBPK model was used to simulate a DDI with MTF, an OCT2/MATE substrate (Kimura et al., 2005), as recommended by the FDA (U.S. Food and Drug Administration, 2019). The association between TQ and MTF is shown in Figure 7 and Supplementary Figure S1, where the negative control curve with MTF alone aligns with the DDI curve across all tested doses. Positive control curves were constructed for the inhibition process by progressively decreasing the maximum reaction rate (Vmax) of the transporters to 0% (Supplementary Tables S10, S11, S12). The main curve (0% activity) was used for the figures. Testing the impact of TQ inhibition on transporters does not reveal changes in the MTF profile aligning with the DDI ratios (Supplementary Tables S5, S6, S7, S8).

A possible explanation for the absence of changes in the PK profile of MTF in this work is the transporter activity in the MTF base model and the extent to which the MTF model predicts changes in the drug’s PK profile related to these transporters. To confirm that the transporters were properly implemented in the MTF model, activity tests were performed. PK-Sim® uses gene expression as a surrogate for protein abundance and activity, including enzymes and transporters (Meyer et al., 2012). Changes in relative protein expression values account for protein abundance and activity in different organs. Therefore, different expression values were assigned to the transporters to assess their functionality (Supplementary Table S9). The simulated curves showed an increase in MTF exposure and a decrease in its elimination as transporter expression levels decreased. This result confirms that the transporters are functionally implemented in the model, as their reduction or inactivation clearly impacts the PK profile of MTF.

To assess whether TQ reached the kidney in relevant amounts, concentration-time profiles of TQ in kidney interstitial and intracellular spaces were generated (Supplementary Figure S2). The TQ profile indicates drug accumulation within the intracellular space, although most of the concentration does not correspond to the unbound fraction, which is responsible for drug activity and interactions (Di, 2021; Smith et al., 2010). The transporters studied were in the basolateral membrane (OCT2) and in the apical (luminal) membrane (MATE1). When correlating the inhibition constants (Ki) used in the model with the predicted kidney concentrations, the expected drug concentration to inhibit MATE1 (1.80 μmol/L or 834.3 ng/mL) was not achieved under any TQ dose regimen or intracellular kidney pH condition. However, the expected concentration to inhibit OCT2 (0.28 μmol/L or 129.78 ng/mL) was achieved at an acidic intracellular kidney pH, as expected given lysosomal sequestration (Schmitt et al., 2019). Still, it is not clear what the drug concentration available for the inhibition process is, as most of it appears to be trapped within the tissue. Tissue concentration is an important piece of information that would really help describe TQ’s distribution. With this type of data, it would be possible to develop a model of subcellular compartments and evaluate the availability of free drug to renal transporters. In addition to this unclear scenario, the inhibition parameters used were derived from the FDA multidisciplinary review and are based on an *in vitro* study using human embryonic kidney 293 (HEK293) cells expressing the transporters (U.S. Food and Drug Administration, 2025). The IC50 and Ki values obtained in this study appear to have been affected by the TQ adsorption ratio on the assay plate, which ranged from 35.9% to 85.2%. A mathematical approach accounting for adsorption was used, and the final IC50 and Ki values were 4–7 times lower than the initial values. These final values were used in the model, with which the estimated ratios between unbound plasma Cmax and IC50 were lower than the cut-off values recommended by the FDA for OCT2 and MATEs transporters (0.1 for OCT2 and 0.02 for MATEs) (U.S. Food and Drug Administration, 2025; U.S. Food and Drug Administration, 2019). Therefore, the absence of the effect of TQ on MTF exposure is consistent with the existing literature on TQ.

## 5 Conclusion

In summary, a whole-body PBPK of TQ has been developed and evaluated in PK-Sim®, including processes that appear to influence the drug’s PK properties, like hepatic clearance. This work serves as a starting point for assessing the ADME profile of TQ, the impact of lysosomal sequestration, and TQ’s behavior in various clinical settings, including liver impairment and pediatric patients. Overall, new studies on TQ ADME are needed to better understand its ADME processes. Such studies would support the development of a more mechanistic model. Even with a few PK data and information regarding TQ, we could assess the lack of DDI across MTF and TQ dose regimens as prolonged administration scenarios for the prophylactic dosing scheme. Our results suggest that TQ does not significantly affect the exposure of OCT2/MATE substrates, such as MTF. Given the available data, these results suggest a low risk of interaction between TQ and OCT2/MATE substrates.

## Data Availability

The original contributions presented in the study are included in the article/Supplementary Material, further inquiries can be directed to the corresponding author.
