# Supplementary material for: Physiologically based pharmacokinetic modeling of tafenoquine and evaluation of transporter-mediated drug–drug interactions
Source: Front Pharmacol. 2026 Jul 17;17:1880776. doi: 10.3389/fphar.2026.1880776 (PMC13423896; doi:10.3389/fphar.2026.1880776)
Supplement: Supplementary file 1 [file Supplementaryfile1.docx]

Supplementary Material

**
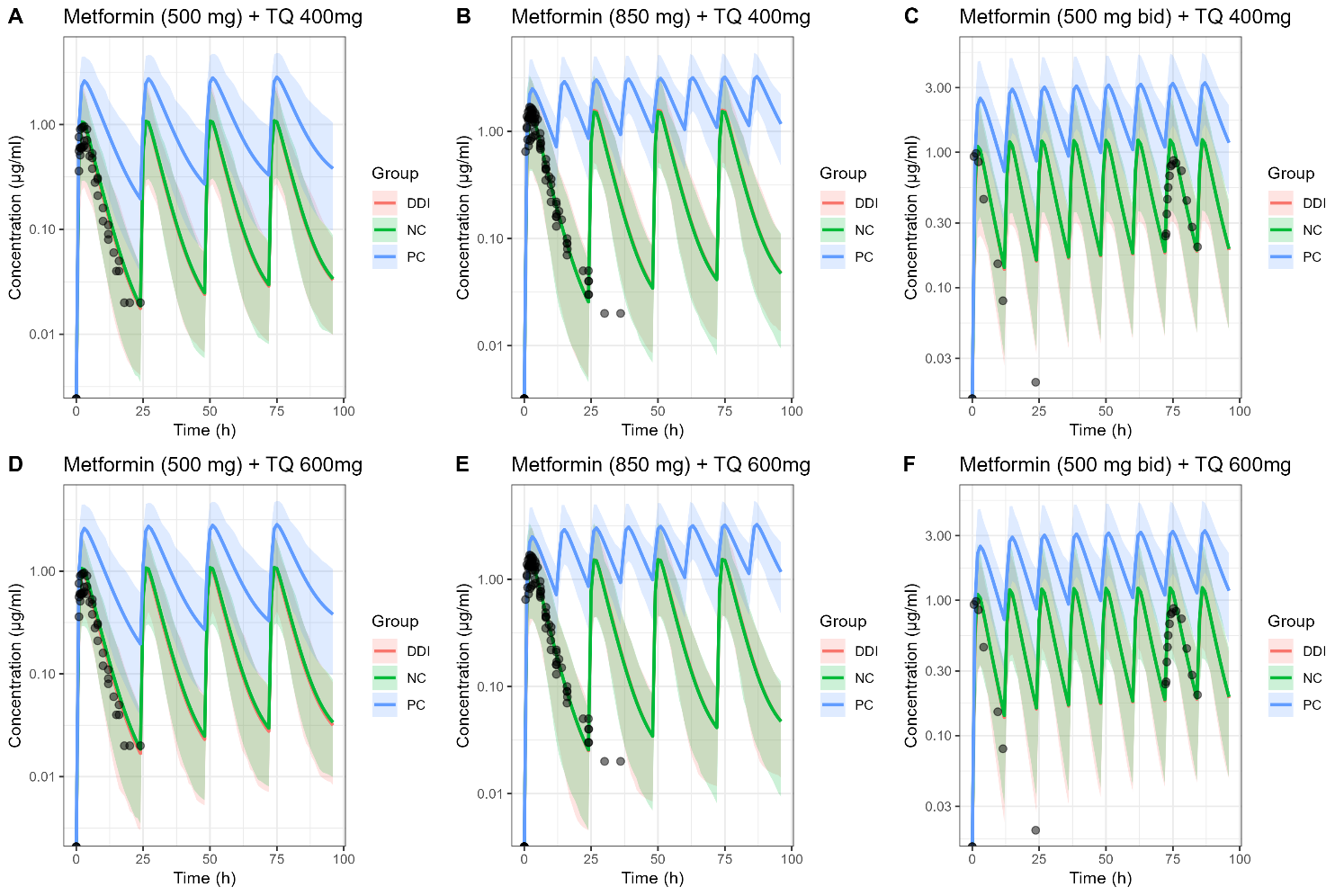
**

**Supplementary Figure 1.** MTF plasma concentration-time profiles (semilogarithmic) co-administered with tafenoquine. Population predictions plasma concentration-time profiles of MTF 500 mg qd with TQ 400 mg (A) and 600 mg (D); MTF 850 mg qd with TQ 400 mg (B) and 600 mg (E); MTF 500 mg bid with TQ 400 mg (C) and 600 mg (F). Observed data are shown as dots, population arithmetic mean is shown as lines, and the shaded area represents the 5^th^ and 95^th^ percentile of simulations divided by negative control (NC), positive control (PC) and coadministration (DDI).

**
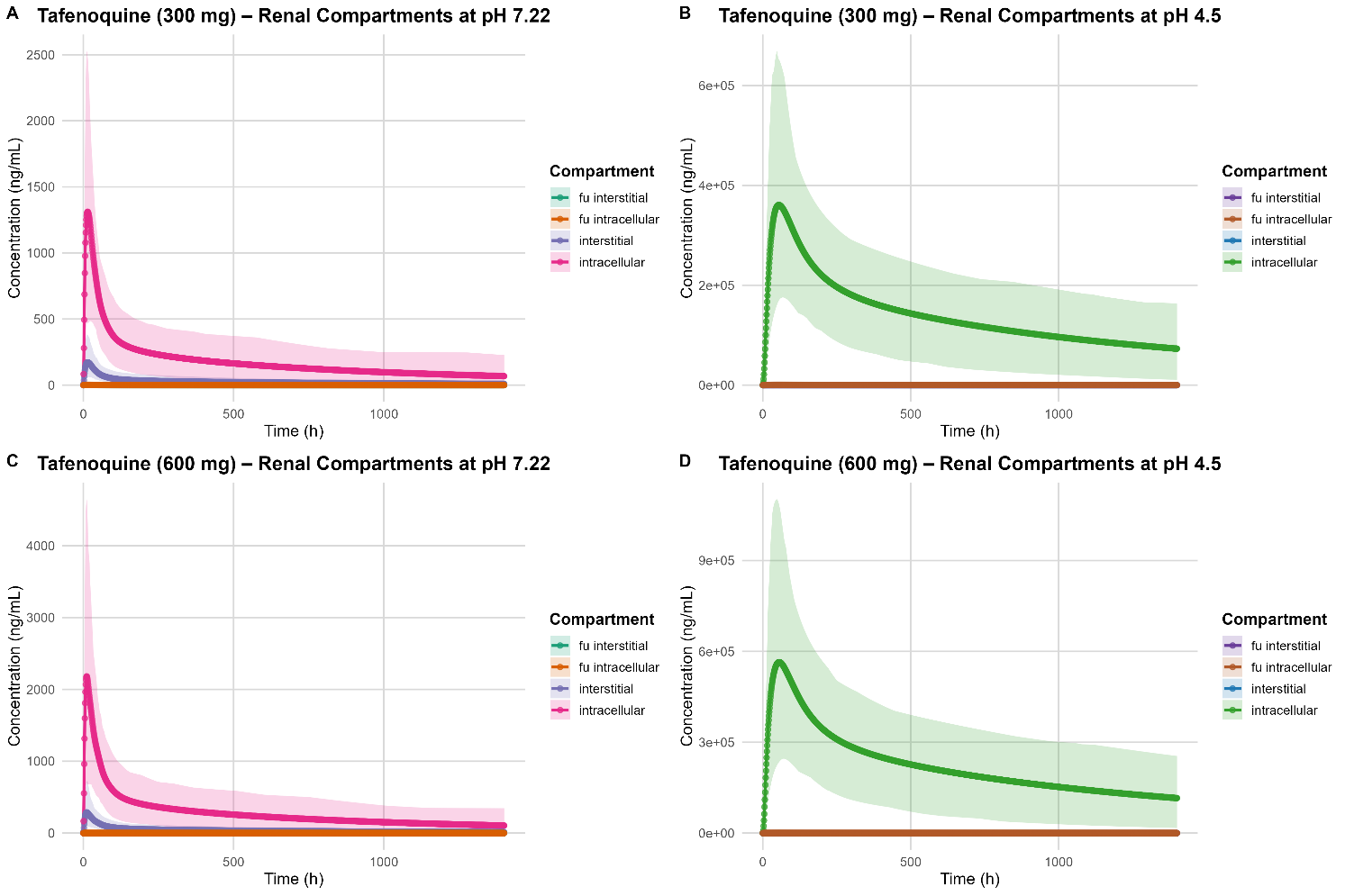
**

**Supplementary Figure 2. TQ renal concentration-time profiles (semilogarithmic) in different renal compartments and pH values. Population predictions renal concentration-time profiles of bound TQ in interstitial and intracellular compartments; and unbound (fu) TQ in interstitial and intracellular compartments after 300 mg (A and B) and 600 mg (C and D) single dose with the default pH (A and C) and acid pH (B and D). Population arithmetic means are shown as lines, the shaded area represents the 5^th^ and 95^th^ percentile of simulations of bound and unbound drug in different compartments of the kidney.**


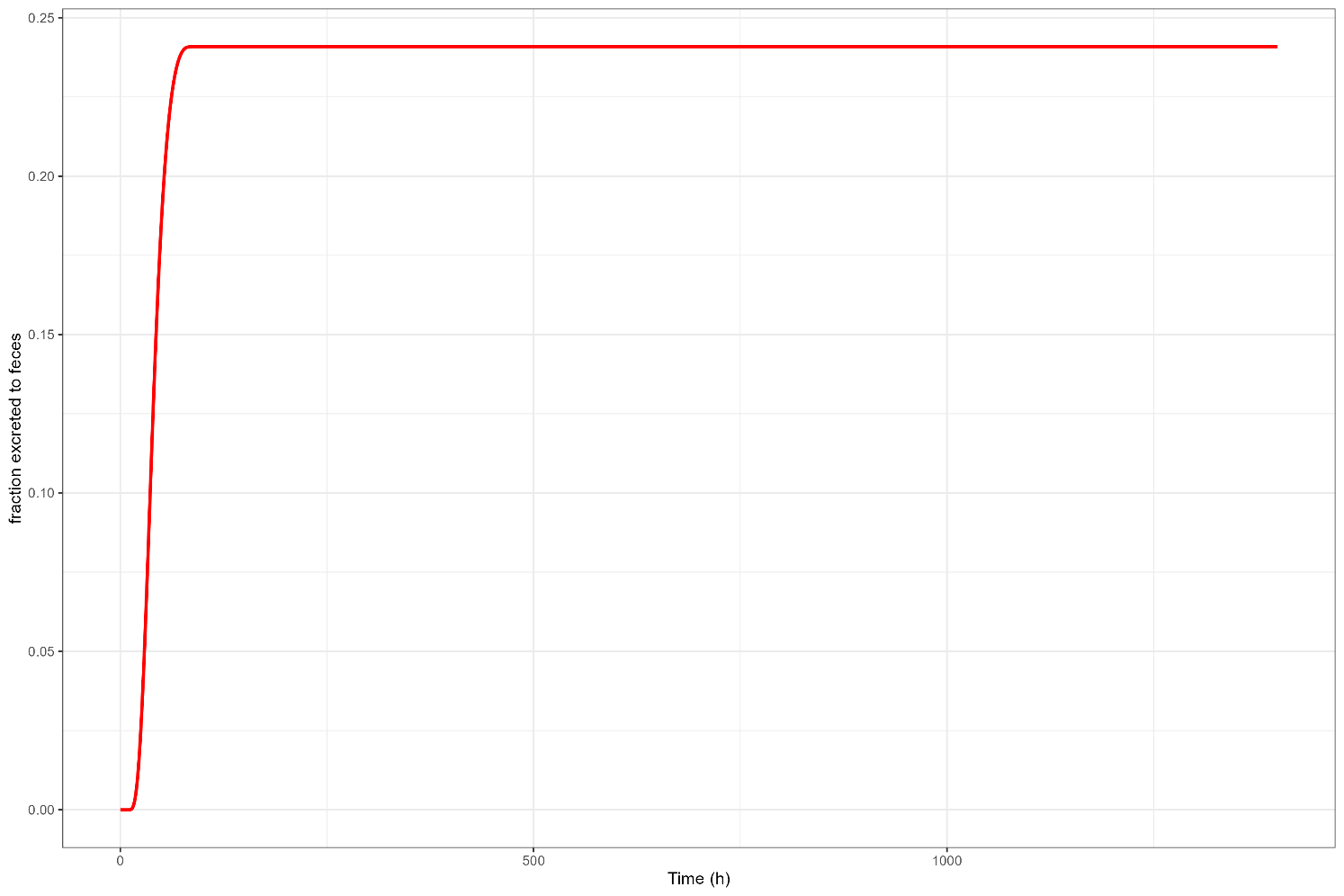


**Supplementary Figure 3.** Simulated fraction of TQ excreted to feces after a 300 mg single dose.

**
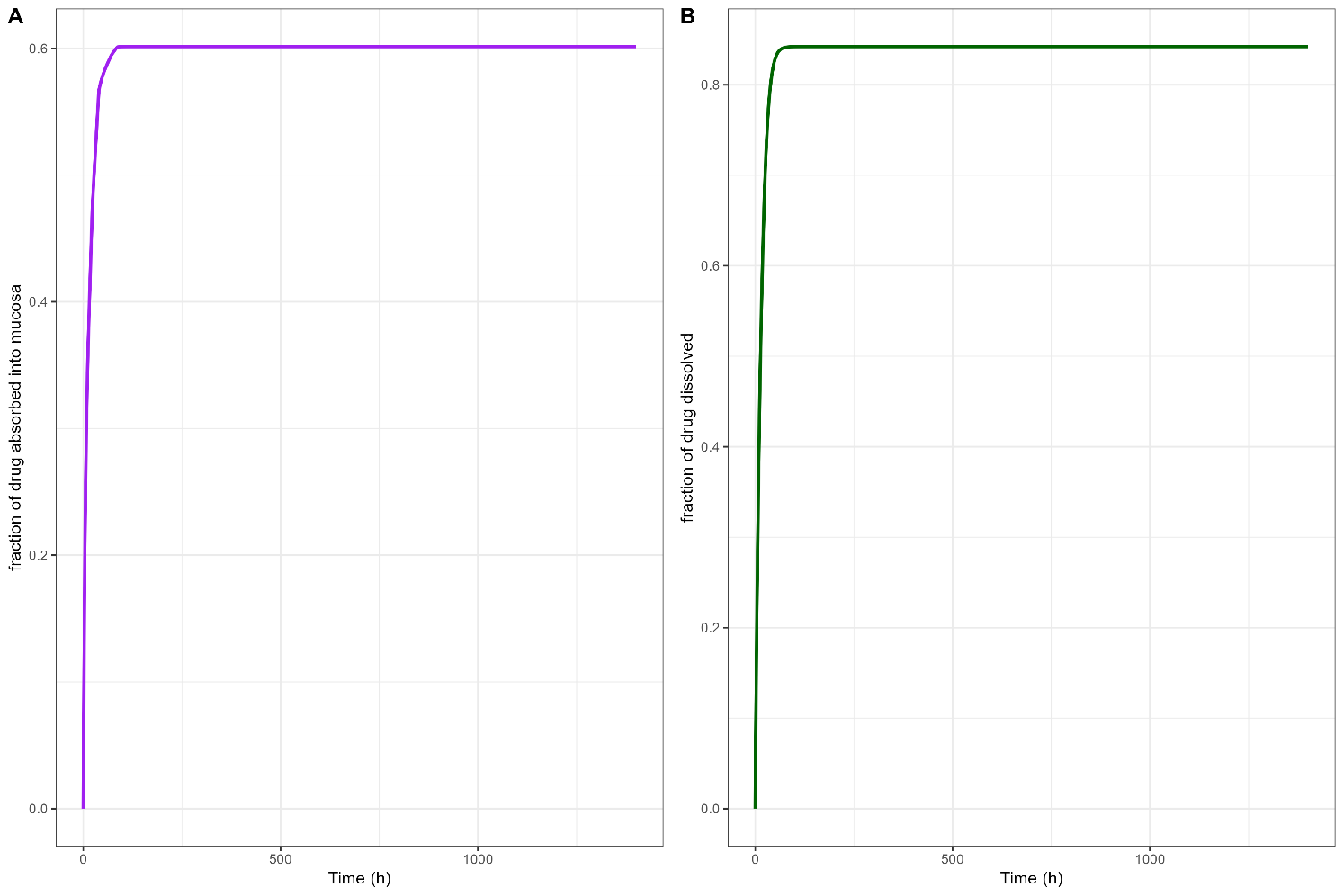
**

**Supplementary Figure 4.** Simulated fractions of TQ in the intestinal lumen after a 300 mg single dose. Fraction of the drug absorbed into the intestinal lumen mucosa (A); fraction of the drug dissolved in the intestinal lumen (B).

**Supplementary Table 1.** Tafenoquine clinical studies.

| **Dose [mg]** | **Route** | **n** | **Men [%]** | **White [%]** | **Age [years]** | **Weight [kg]** | **Height [cm]** | **BMI [kg/m²]** | **Dataset** | **Reference** |
| --- | --- | --- | --- | --- | --- | --- | --- | --- | --- | --- |
| 200 | po, tablet | 3 | 67 | 67 | 40 (28-53) | 87 (61-95) | - | 29 (26-30) | training | (1) |
| 300 | po, tablet | 4 | 25 | 100 | 34 (20-45) | 81 (71-108) | - | 27 (27-31) | training | (1) |
| 300 | po, tablet | 24 | 75 | 8 | 35.8 | 76.3 | 173.6 | - | test | (2) |
| 400 | po, tablet | 2 | 0 | 100 | 31 (20-41) | 52 (51-53) | - | 20 (20-21) | training | (1) |
| 600 | po, tablet | 3 | 100 | 100 | 23 (19-23) | 67 (65-100) | - | 21 (20-30) | training | (1) |
| 600 | po, capsule | 48 | 100 | 42 | 27.8 | 76.1 | 176.4 | - | test | (3) |
| 600 | po, capsule | 18 | 72 | - | 22 (18-41) | 57 (44-93) | - | - | test | (4) |
| 200 | po, tablet | 12 | 33 | 100 | 25.3 (20-30) | 69.8 (56-97.7) | - | 23.2 (18.6-30.2) | test | (5) |
| 200 | po, tablet | 6 | 0 | - | 23.3 (19-28) | - | - | - | test | (6) |
| 300 | po, tablet | 6 | 0 | - | 31.8 (24-36) | - | - | - | test | (6) |
| 450 | po, tablet | 20 | 60 | 55 | 27 | - | - | - | test | (7) |

**Supplementary Table 2.** Statistical Evaluation of TQ Model-Based Predictions Across Studies.

|  | | | **AUC** | | | **C_max_** | | |
| --- | --- | --- | --- | --- | --- | --- | --- | --- |
| **Reference** | **Dose [mg]** | **MRD** | **Pred [ng*h/mL]** | **Obs [ng*h/mL]** | **Pred/Obs** | **Pred [ng/mL]** | **Obs [ng/mL]** | **Pred/Obs** |
| (1) | 200 | 1.27 | 70973.19 | 48019.1 | 1.48 | 342.04 | 141 | 2.43 |
| (1) | 300 | 1.23 | 102713.2 | 64224.15 | 1.60 | 478.22 | 210.39 | 2.27 |
| (2) | 300 | 1.36 | 102713.2 | 120819.6 | 0.85 | 478.22 | 194.20 | 2.46 |
| (1) | 400 | 1.14 | 127715.3 | 105080.61 | 1.22 | 583.03 | 328.22 | 1.78 |
| (1) | 600 | 1.15 | 136557.9 | 118908.64 | 1.15 | 761.18 | 346.67 | 2.20 |
| (3) | 600 | 1.13 | 136557.9 | 90961.8 | 1.50 | 761.18 | 260.0 | 2.93 |
| (4) | 600 | 1.11 | 136557.9 | 167747.79 | 0.81 | 761.18 | 646.12 | 1.18 |
| (5) | 200 | 1.24 | 199280 | 125289 | 1.59 | 572.64 | 188.44 | 3.04 |
| (6) | 200 | 1.11 | 70973.19 | 68351.86 | 1.04 | 342.04 | 171.01 | 2.0 |
| (6) | 300 | 1.07 | 102713.2 | 123703 | 0.83 | 478.22 | 291.90 | 1.64 |
| (7) | 450 | 1.72 | 227582.3 | 273597 | 0.83 | 940.50 | 675.81 | 1.39 |
| Overall MRD | | 1.23 |  | | | | | |
| Overall GMFE | |  | 1.19 | | | 2.0 | | |

**Supplementary Table 3. Predicted Impact of Hepatic Impairment on TQ profile.**

| **Parameters** | **Healthy** | | **Child-Pugh A** | | **Child-Pugh B** | | **Child-Pugh C** | |
| --- | --- | --- | --- | --- | --- | --- | --- | --- |
|  | **TQ 300 mg** | **TQ prophy** | **TQ 300 mg** | **TQ prophy** | **TQ 300 mg** | **TQ prophy** | **TQ 300 mg** | **TQ prophy** |
| **AUC_0-tlast_ [ng*h/mL]** | **102713.2** | **270642.8** | **116758.6** | **316875.5** | **132226.9** | **350569.8** | **106637.6** | **287376.6** |
| **AUC extrapolated [%]** | **25.78** | **28.42** | **39.60** | **41.04** | **46.36** | **44.14** | **39.87** | **39.77** |
| **AUC_0-inf_ [ng*h/mL]** | **138381.1** | **378075** | **193311.5** | **537480.3** | **246509.1** | **627554.6** | **177346.4** | **477157.9** |
| **Cl [L/h]** | **2.92** | **2.82** | **2.57** | **1.71** | **2.27** | **1.51** | **2.81** | **1.88** |
| **ke [*10^-4^ h^-1^]** | **8.2** | **8.0** | **5.85** | **5.93** | **4.80** | **5.38** | **5.72** | **6.04** |
| **Half-time [h]** | **843.42** | **852.36** | **1184.71** | **1168.25** | **1444.89** | **1288.08** | **1212.05** | **1147.46** |

**Supplementary Table 4.** Statistical Evaluation of MTF Model-Based Predictions Across Studies.

|  | | | **AUC** | | | **C_max_** | | |
| --- | --- | --- | --- | --- | --- | --- | --- | --- |
| **Reference** | **Dose [mg]** | **MRD** | **Pred [ng*h/mL]** | **Obs [ng*h/mL]** | **Pred/Obs** | **Pred [ng/mL]** | **Obs [ng/mL]** | **Pred/Obs** |
| (8) | 500 24q | 1.66 | 7.02 | 4.44 | 1.58 | 1.09 | 0.63 | 1.73 |
| (9) | 500 24q | 1.51 | 7.02 | 5.01 | 1.40 | 1.09 | 0.71 | 1.54 |
| (10) | 500 12q | 1.19 | 6.25 | 5.28 | 1.18 | 1.11 | 0.98 | 1.13 |
| (11) | 500 12q | 2.89 | 6.25 | 6.55 | 0.95 | 1.11 | 0.87 | 1.28 |
| (12) | 850 24q | 1.41 | 8.85 | 7.81 | 1.13 | 1.50 | 1.36 | 1.10 |
| (13) | 850 24q | 1.70 | 8.85 | 10.98 | 0.81 | 1.50 | 1.69 | 0.89 |
| (14) | 850 24q | 1.30 | 8.85 | 8.36 | 1.06 | 1.50 | 1.57 | 0.96 |
| (15) | 850 24q | 1.17 | 8.85 | 9.67 | 0.92 | 1.50 | 1.33 | 1.13 |
| (16) | 850 24q | 3.57 | 8.85 | 11.59 | 0.76 | 1.50 | 1.67 | 0.90 |
| (17) | 850 24q | 1.21 | 8.85 | 7.49 | 1.18 | 1.50 | 0.92 | 1.63 |
| Overall MRD | | 1.76 |  | | | | | |
| Overall GMFE | |  | 0.96 | | | 1.21 | | |

**Supplementary Table 5.** Predicted and Observed Tafenoquine-Metformin DDI AUC Ratios and DDI C_max_ ratios.

| **Perpetrator** | **Victim** |  | **AUC [µg*h/mL]** | | | | **C_max_ [µg/mL]** | | |  |
| --- | --- | --- | --- | --- | --- | --- | --- | --- | --- | --- |
| Tafenoquine | Metformin | Genotype | **Coadmin** | **NC** | **Coadmin/NC** | | **Coadmin** | **NC** | **Coadmin/NC** | **Reference** |
| 300 mg, po, qd | 500 mg, po, qd | - | 6.10 | 7.02 | 0.87 | | 0.95 | 1.09 | 0.87 | (8,9) |
| 300 mg, po, qd | 500 mg, po, bid | - | 13.06 | 6.25 | 2.09 | | 1.19 | 1.11 | 1.08 | (10,11) |
| 300 mg, po, qd | 850 mg, po, qd | - | 9.00 | 8.85 | 1.02 | | 1.35 | 1.50 | 0.90 | (12,13,14,15,16,17) |
| 400 mg, po, qd | 500 mg, po, qd | - | 6.58 | 7.02 | | 0.94 | 1.02 | 1.09 | 0.94 | (8,9) |
| 400 mg, po, qd | 500 mg, po, bid | - | 12.80 | 6.25 | | 2.05 | 1.15 | 1.11 | 1.04 | (10,11) |
| 400 mg, po, qd | 850 mg, po, qd | - | 9.75 | 8.85 | | 1.10 | 1.48 | 1.50 | 0.98 | (12,13,14,15,16,17) |
| 600 mg, po, qd | 500 mg, po, qd | - | 6.37 | 7.02 | | 0.91 | 1.0 | 1.09 | 0.92 | (8,9) |
| 600 mg, po, qd | 500 mg, po, bid | - | 12.79 | 6.25 | | 2.05 | 1.15 | 1.11 | 1.04 | (10,11) |
| 600 mg, po, qd | 850 mg, po, qd | - | 9.59 | 8.85 | | 1.08 | 1.44 | 1.50 | 0.96 | (12,13,14,15,16,17) |
| 200 mg, po, prophy | 500 mg, po, qd | - | 6.60 | 7.02 | | 0.94 | 1.00 | 1.09 | 0.93 | (8,9) |
| 200 mg, po, prophy | 500 mg, po, bid | - | 12.75 | 6.25 | | 2.04 | 1.18 | 1.11 | 1.06 | (10,11) |
| 200 mg, po, prophy | 850 mg, po, qd | - | 9.93 | 8.85 | | 1.12 | 1.51 | 1.50 | 1.00 | (12,13,14,15,16,17) |
| Overall ratios | | | 1.35 | | | | 0.98 | | |  |

Coadmin: coadministration; NC: negative control

**Supplementary Table 6.** Impact of Removing TQ-Mediated OCT2 Inhibition on MTF Profile.

| **Parameters** | **MTF 500 mg** | | | | | **MTF 500 mg bid** | | | | | **MTF 850 mg** | | | | |
| --- | --- | --- | --- | --- | --- | --- | --- | --- | --- | --- | --- | --- | --- | --- | --- |
|  | **Control** | **TQ 300 mg** | **TQ 400 mg** | **TQ 600 mg** | **TQ prophy** | **Control** | **TQ 300 mg** | **TQ 400 mg** | **TQ 600 mg** | **TQ prophy** | **Control** | **TQ 300 mg** | **TQ 400 mg** | **TQ 600 mg** | **TQ prophy** |
| AUC_0-tlast_ [µg*h/mL] | 7.02 | 6.23 | 6.23 | 6.23 | 6.23 | 6.25 | 5.71 | 5.71 | 5.71 | 5.71 | 8.85 | 8.98 | 8.98 | 8.98 | 8.98 |
| AUC extrapolated [%] | 1.82 | 8.92 | 8.92 | 8.92 | 8.92 | 9.43 | 9.65 | 9.65 | 9.65 | 9.65 | 9.08 | 8.55 | 8.55 | 8.55 | 8.55 |
| AUC_0-inf_ [µg*h/mL] | 7.15 | 6.84 | 6.84 | 6.84 | 6.84 | 6.90 | 6.32 | 6.32 | 6.32 | 6.32 | 9.73 | 9.82 | 9.82 | 9.82 | 9.82 |
| C_max_ [µg/mL] | 1.09 | 1.09 | 1.09 | 1.09 | 1.09 | 1.11 | 0.97 | 0.97 | 0.97 | 0.97 | 1.50 | 1.54 | 1.54 | 1.54 | 1.54 |
| T_max_ [h] | 2.5 | 2 | 2 | 2 | 2 | 2 | 2 | 2 | 2 | 2 | 2 | 2 | 2 | 2 | 2 |
| ke [h^-1^] | 0.14 | 0.23 | 0.23 | 0.23 | 0.23 | 0.22 | 0.22 | 0.22 | 0.22 | 0.22 | 0.23 | 0.23 | 0.23 | 0.23 | 0.23 |
| Half-time [h] | 4.84 | 3.08 | 3.08 | 3.08 | 3.08 | 3.20 | 3.12 | 3.12 | 3.12 | 3.12 | 3.05 | 2.96 | 2.96 | 2.96 | 2.96 |

**Supplementary Table 7.** Impact of Removing TQ-Mediated MATE1 Inhibition on MTF Profile.

| **Parameters** | **MTF 500 mg** | | | | | **MTF 500 mg bid** | | | | | **MTF 850 mg** | | | | | |
| --- | --- | --- | --- | --- | --- | --- | --- | --- | --- | --- | --- | --- | --- | --- | --- | --- |
|  | **Control** | **TQ 300 mg** | **TQ 400 mg** | **TQ 600 mg** | **TQ prophy** | **Control** | **TQ 300 mg** | **TQ 400 mg** | **TQ 600 mg** | **TQ prophy** | **Control** | **TQ 300 mg** | **TQ 400 mg** | **TQ 600 mg** | **TQ prophy** |  |
| AUC_0-tlast_ [µg*h/mL] | 7.02 | 6.22 | 6.22 | 6.22 | 6.22 | 6.25 | 5.97 | 5.97 | 5.97 | 5.97 | 8.85 | 8.79 | 8.79 | 8.79 | 8.79 |  |
| AUC extrapolated [%] | 1.82 | 8.80 | 8.80 | 8.80 | 8.80 | 9.43 | 9.55 | 9.55 | 9.55 | 9.55 | 9.08 | 9.10 | 9.10 | 9.10 | 9.10 |  |
| AUC_0-inf_ [µg*h/mL] | 7.15 | 6.82 | 6.82 | 6.82 | 6.82 | 6.90 | 6.60 | 6.60 | 6.60 | 6.60 | 9.73 | 9.67 | 9.67 | 9.67 | 9.67 |  |
| C_max_ [µg/mL] | 1.09 | 1.08 | 1.08 | 1.08 | 1.08 | 1.11 | 1.02 | 1.02 | 1.02 | 1.02 | 1.50 | 1.50 | 1.50 | 1.50 | 1.50 |  |
| T_max_ [h] | 2.5 | 2 | 2 | 2 | 2 | 2 | 2 | 2 | 2 | 2 | 2 | 2 | 2 | 2 | 2 |  |
| ke [h^-1^] | 0.14 | 0.23 | 0.23 | 0.23 | 0.23 | 0.22 | 0.22 | 0.22 | 0.22 | 0.22 | 0.23 | 0.23 | 0.23 | 0.23 | 0.23 |  |
| Half-time [h] | 4.84 | 3.03 | 3.03 | 3.03 | 3.03 | 3.20 | 3.13 | 3.13 | 3.13 | 3.13 | 3.05 | 3.05 | 3.05 | 3.05 | 3.05 |  |

**Supplementary Table 8.** Impact of Removing TQ-Mediated OCT2 and MATE1 Inhibition on MTF Profile.

| **Parameters** | **MTF 500 mg** | | | | | **MTF 500 mg bid** | | | | | **MTF 850 mg** | | | | |
| --- | --- | --- | --- | --- | --- | --- | --- | --- | --- | --- | --- | --- | --- | --- | --- |
|  | **Control** | **TQ 300 mg** | **TQ 400 mg** | **TQ 600 mg** | **TQ prophy** | **Control** | **TQ 300 mg** | **TQ 400 mg** | **TQ 600 mg** | **TQ prophy** | **Control** | **TQ 300 mg** | **TQ 400 mg** | **TQ 600 mg** | **TQ prophy** |
| AUC_0-tlast_ [µg*h/mL] | 7.02 | 6.22 | 6.22 | 6.22 | 6.22 | 6.25 | 5.97 | 5.97 | 5.97 | 5.97 | 8.85 | 8.79 | 8.79 | 8.79 | 8.79 |
| AUC extrapolated [%] | 1.82 | 8.80 | 8.80 | 8.80 | 8.80 | 9.43 | 9.55 | 9.55 | 9.55 | 9.55 | 9.08 | 9.10 | 9.10 | 9.10 | 9.10 |
| AUC_0-inf_ [µg*h/mL] | 7.15 | 6.82 | 6.82 | 6.82 | 6.82 | 6.90 | 6.60 | 6.60 | 6.60 | 6.60 | 9.73 | 9.67 | 9.67 | 9.67 | 9.67 |
| C_max_ [µg/mL] | 1.09 | 1.08 | 1.08 | 1.08 | 1.08 | 1.11 | 1.02 | 1.02 | 1.02 | 1.02 | 1.50 | 1.50 | 1.50 | 1.50 | 1.50 |
| T_max_ [h] | 2.5 | 2 | 2 | 2 | 2 | 2 | 2 | 2 | 2 | 2 | 2 | 2 | 2 | 2 | 2 |
| ke [h^-1^] | 0.14 | 0.23 | 0.23 | 0.23 | 0.23 | 0.22 | 0.22 | 0.22 | 0.22 | 0.22 | 0.23 | 0.23 | 0.23 | 0.23 | 0.23 |
| Half-time [h] | 4.84 | 3.03 | 3.03 | 3.03 | 3.03 | 3.20 | 3.12 | 3.12 | 3.12 | 3.12 | 3.05 | 3.05 | 3.05 | 3.05 | 3.05 |

**Supplementary Table 9.** Effect of OCT2 Expression on MTF PK Parameters.

| **Parameters** | **MTF 500 mg** | | | | **MTF 500 mg bid** | | | | **MTF 850 mg** | | | |
| --- | --- | --- | --- | --- | --- | --- | --- | --- | --- | --- | --- | --- |
|  | **Control** | **50%** | **25%** | **0%** | **Control** | **50%** | **25%** | **0%** | **Control** | **50%** | **25%** | **0%** |
| AUC_0-tlast_ [µg*h/mL] | 7.02 | 5.38 | 6.12 | 13.02 | 6.25 | 5.38 | 6.12 | 13.02 | 8.85 | 7.7 | 8.76 | 18.44 |
| AUC extrapolated [%] | 1.82 | 8.60 | 8.79 | 17.18 | 9.43 | 8.19 | 8.79 | 17.18 | 9.08 | 8.22 | 8.84 | 17.42 |
| AUC_0-inf_ [µg*h/mL] | 7.15 | 5.89 | 6.71 | 15.72 | 6.90 | 5.86 | 6.71 | 15.72 | 9.73 | 8.39 | 9.61 | 22.33 |
| C_max_ [µg/mL] | 1.09 | 0.89 | 1 | 1.82 | 1.11 | 0.89 | 1 | 1.82 | 1.50 | 1.28 | 1.43 | 2.57 |
| T_max_ [h] | 2.5 | 3 | 3 | 3 | 2 | 3 | 3 | 3 | 2 | 3 | 3 | 3 |
| ke [h^-1^] | 0.14 | 0.25 | 0.24 | 0.18 | 0.22 | 0.25 | 0.24 | 0.18 | 0.23 | 0.25 | 0.24 | 0.18 |
| Half-time [h] | 4.84 | 2.79 | 2.87 | 3.85 | 3.20 | 2.79 | 2.87 | 3.85 | 3.05 | 2.79 | 2.87 | 3.87 |

**Supplementary Table 10.** Effect of OCT2 Vmax Variations on Metformin PK Parameters.

| **Parameters** | **MTF 500 mg** | | | | **MTF 500 mg bid** | | | | **MTF 850 mg** | | | |
| --- | --- | --- | --- | --- | --- | --- | --- | --- | --- | --- | --- | --- |
|  | **Control** | **50%** | **25%** | **0%** | **Control** | **50%** | **25%** | **0%** | **Control** | **50%** | **25%** | **0%** |
| AUC_0-tlast_ [µg*h/mL] | 7.02 | 5.39 | 6.13 | 13.13 | 6.25 | 5.38 | 6.13 | 13.13 | 8.85 | 7.71 | 8.78 | 18.59 |
| AUC extrapolated [%] | 1.82 | 8.00 | 8.64 | 16.42 | 9.43 | 8.09 | 8.61 | 16.42 | 9.08 | 8.09 | 8.72 | 16.63 |
| AUC_0-inf_ [µg*h/mL] | 7.15 | 5.86 | 6.71 | 15.71 | 6.90 | 5.86 | 6.71 | 15.71 | 9.73 | 8.39 | 9.61 | 22.30 |
| C_max_ [µg/mL] | 1.09 | 0.89 | 1.00 | 1.86 | 1.11 | 0.89 | 1.00 | 1.86 | 1.50 | 1.28 | 1.43 | 2.63 |
| T_max_ [h] | 2.5 | 3 | 3 | 3 | 2 | 3 | 3 | 3 | 2 | 3 | 3 | 3 |
| ke [h^-1^] | 0.14 | 0.25 | 0.24 | 0.18 | 0.22 | 0.25 | 0.24 | 0.18 | 0.23 | 0.25 | 0.24 | 0.18 |
| Half-time [h] | 4.84 | 2.78 | 2.86 | 3.78 | 3.20 | 2.79 | 2.86 | 3.78 | 3.05 | 2.78 | 2.86 | 3.80 |

**Supplementary Table 11.** Effect of MATE1 Vmax Variations on Metformin PK Parameters.

| **Parameters** | **MTF 500 mg** | | | | **MTF 500 mg bid** | | | | **MTF 850 mg** | | | |
| --- | --- | --- | --- | --- | --- | --- | --- | --- | --- | --- | --- | --- |
|  | **Control** | **50%** | **25%** | **0%** | **Control** | **50%** | **25%** | **0%** | **Control** | **50%** | **25%** | **0%** |
| AUC_0-tlast_ [µg*h/mL] | 7.02 | 4.96 | 4.97 | 13.82 | 6.25 | 4.96 | 4.97 | 13.82 | 8.85 | 7.10 | 7.12 | 19.59 |
| AUC extrapolated [%] | 1.82 | 7.71 | 7.72 | 25.95 | 9.43 | 7.71 | 7.72 | 25.95 | 9.08 | 7.80 | 7.82 | 26.08 |
| AUC_0-inf_ [µg*h/mL] | 7.15 | 5.37 | 5.39 | 18.67 | 6.90 | 5.37 | 5.39 | 18.67 | 9.73 | 7.70 | 7.73 | 26.50 |
| C_max_ [µg/mL] | 1.09 | 0.83 | 0.83 | 1.70 | 1.11 | 0.83 | 0.83 | 1.70 | 1.50 | 1.19 | 1.19 | 2.41 |
| T_max_ [h] | 2.5 | 3 | 3 | 3 | 2 | 3 | 3 | 3 | 2 | 3 | 3 | 4 |
| ke [h^-1^] | 0.14 | 0.25 | 0.25 | 0.14 | 0.22 | 0.25 | 0.25 | 0.14 | 0.23 | 0.25 | 0.25 | 0.14 |
| Half-time [h] | 4.84 | 2.74 | 2.74 | 4.82 | 3.20 | 2.74 | 2.74 | 4.82 | 3.05 | 2.74 | 2.75 | 4.83 |

**Supplementary Table 12.** Effect of OCT2 and MATE1 Vmax Variations on Metformin PK Parameters.

| **Parameters** | **MTF 500 mg** | | | | **MTF 500 mg bid** | | | | **MTF 850 mg** | | | |
| --- | --- | --- | --- | --- | --- | --- | --- | --- | --- | --- | --- | --- |
|  | **Control** | **50%** | **25%** | **0%** | **Control** | **50%** | **25%** | **0%** | **Control** | **50%** | **25%** | **0%** |
| AUC_0-tlast_ [µg*h/mL] | 7.02 | 5.40 | 6.20 | 14.95 | 6.25 | 5.40 | 6.20 | 14.95 | 8.85 | 7.73 | 8.87 | 21.09 |
| AUC extrapolated [%] | 1.82 | 8.00 | 8.67 | 19.09 | 9.43 | 8.00 | 8.67 | 19.09 | 9.08 | 8.11 | 8.78 | 19.33 |
| AUC_0-inf_ [µg*h/mL] | 7.15 | 5.87 | 6.79 | 18.47 | 6.90 | 5.87 | 6.79 | 18.47 | 9.73 | 8.41 | 9.72 | 26.12 |
| C_max_ [µg/mL] | 1.09 | 0.90 | 1.01 | 2.04 | 1.11 | 0.90 | 1.01 | 2.04 | 1.50 | 1.28 | 1.45 | 2.87 |
| T_max_ [h] | 2.5 | 3 | 3 | 3 | 2 | 3 | 3 | 3 | 2 | 3 | 3 | 3 |
| ke [h^-1^] | 0.14 | 0.25 | 0.24 | 0.17 | 0.22 | 0.25 | 0.24 | 0.17 | 0.23 | 0.25 | 0.24 | 0.17 |
| Half-time [h] | 4.84 | 2.78 | 2.87 | 4.11 | 3.20 | 2.78 | 2.87 | 4.11 | 3.05 | 2.79 | 2.87 | 4.13 |

**References**

1. Barber, B.E., Abd-Rahman, A.N., Webster, R., Potter, A.J., Llewellyn, S., Marquart, L., Sahai, N., Leelasena, I., Birrell, G.W., Edstein, M.D., Shanks, G.D., Wesche, D., Moehrle, J.J., McCarthy, J.S. (2023). Characterizing the blood-stage antimalarial activity of tafenoquine in healthy volunteers experimentally infected with Plasmodium falciparum. Clin Infect Dis, 76(11). doi: 10.1093/cid/ciad075
2. Green, J.A., Mohamed, K., Goyal, N., Bouhired, S., Hussaini, A., Jones, S.W., Koh, G.C.K, Kostov, I., Taylor, M., Wolstenholm, A., Duparc, A. (2016). Pharmacokinetic interactions between tafenoquine and dihydroartemisinin-piperaquine or artemether-lumefantrine in healthy adult subjects. Antimicrob Agents Chemother, 60(12). doi: 10.1128/AAC.01588-16
3. Brueckner, R.P., Lasseter, K.C., Lin, E.T., Schuster, B.G. (1998). First-time-in-humans safety and pharmacokinetics of WR 238605, a new antimalarial. Am J Trop Med Hyg, 58(5). doi: 10.4269/ajtmh.1998.58.645
4. Walsh, D.S., Wilairatana, P., Tang, D.B., Heppner Jr, D.G., Brewer, T.G., Krudsood, S., Silachamroon, U., Phumratanaprapin, W., Siriyanonda, D., Looareesuwan, S. (2004). Randomized Trial of 3-Dose Regimens of Tafenoquine (WR238605) versus Low-Dose Primaquine for Preventing Plasmodium vivax Malaria Relapse. Clin Infect Dis, 39(8). doi: 10.1086/424508
5. McCarthy, J.S., Smith, B., Reid, M., Berman, J., Marquart, L., Dobblin, C., West, L., Read, L.T., Dow, G.S. (2019). Blood Schizonticidal Activity and Safety of Tafenoquine When Administered as Chemoprophylaxis to Healthy, Nonimmune Participants Followed by Blood Stage *Plasmodium falciparum* Challenge: A Randomized, Double-blind, Placebo-controlled Phase 1b Study. Clin Infec Dis, 69(3). doi: 10.1093/cid/ciy939
6. Rueangweerayut, R., Bancone, G., Harrell, E.J., Beelen, A.P., Kongpatanakul, S., Möhrle, J.J., Rousell, V., Mohamed, K., Qureshi, A., Narayan, S., Yubon, N., Miller, A., Nosten, F.H., Luzzatto, L., Duparc, S., Kleim, J-P., Green, J.A. Hemolytic Potential of Tafenoquine in Female Volunteers Heterozygous for Glucose-6-Phosphate Dehydrogenase (G6PD) Deficiency (G6PD Mahidol Variant) versus G6PD-Normal Volunteers. Am J Trop Med Hyg, 97(3). doi: 10.4269/ajtmh.16-0779
7. Miller, A.K., Harrell, E., Ye, L., Baptiste-Brown, S., Kleim, J-P., Ohrt, C., Duparc, S., Möhrle, J.J., Webster, A., Stinnet, S., Hughes, A., Griffith, S., Beelen, A.P. Pharmacokinetic interactions and safety evaluations of coadministered tafenoquine and chloroquine in healthy subjects. Br J Clin Pharmacol, 76(6). doi: 10.1111/bcp.12160
8. Caillé, G., Lacasse, Y., Raymond, M., Landriault, H., Perrota, M., Picirilli, G., Thiffault, J., Spénard, J. (1993). Bioavailability of metformin in tablet form using a new high pressure liquid chromatography assay method. Biopharm Drug Dispos, 14(3). doi: 10.1002/bdd.2510140308
9. Gusler, G., Gorsline, J., Levy, G., Zhang, S.Z., Weston, I.E., Naret, D., Berner, B. (2001). Pharmacokinetics of Metformin Gastric-Retentive Tablets in Healthy Volunteers. J Clin Pharmacol, 41(6). doi: 10.1177/00912700122010546
10. Tucker, G.T., Casey, C., Phillips, P.J., Connor, H., Ward, J.D., Woods, H.F. (1981). Metformin kinetics in healthy subjects and in patients with diabetes mellitus. Br J Clin Pharmacol, 12(2). doi: 10.1111/j.1365-2125.1981.tb01206.x
11. Di Cicco, R.A., Allen, A., Carr, A., Fowles, S., Jorkasky, D.K., Freed, M.I. (2000). Rosiglitazone does not alter the pharmacokinetics of metformin. J Clin Pharmacol, 40(11). doi: 10.1177/009127000004001113
12. Chen, Y., Li, S., Brown, C., Cheatham, S., Castro, R.A., Leabman, M.K., Urban, T.J., Chen, L., Yee, S.W., Choi, J.H., Huang, Y., Brett, C.M., Burchard, E.G., Giacomini, K.M. (2009). Effect of genetic variation in the organic cation transporter 2 on the renal elimination of metformin. Pharmacogenet Genomics, 19(7). doi: 10.1097/FPC.0b013e32832cc7e9
13. Morrissey, K.M., Stocker, S.L., Chen, E.C., Castro, R.A., Brett, C.M., Giacomini, K.M. (2016). The Effect of Nizatidine, a MATE2K Selective Inhibitor, on the Pharmacokinetics and Pharmacodynamics of Metformin in Healthy Volunteers. Clin Pharmacokinet, 55(4). doi: 10.1007/s40262-015-0332-9
14. Robert, F., Fendri, S., Hary, L., Lacroix, C., Andréjak, M., Lalau, J.D. (2003). Kinetics of plasma and erythrocyte metformin after acute administration in healthy subjects. Diabetes Metab, 29(3). doi: 10.1016/s1262-3636(07)70037-x
15. Sambol, N.C., Chiang, J., Lin, E.T., Gooman, A.M., Liu, C.Y., Benet, L.Z., Cogan, M.G. (1995). Kidney Function and Age Are Both Predictors of Pharmacokinetics of Metformin. J Clin Pharmacol, 35(11). doi: 10.1002/j.1552-4604.1995.tb04033.x
16. Sambol, N.C., Brookes, L.G., Chiang, J., Goodman, A.M., Lin, E.T., Liu, C.Y., Benet, L.Z. (1996a). Food intake and dosage level, but not tablet vs solution dosage form, affect the absorption of metformin HCl in man. Br J Clin Pharmacol, 42(4). doi: 10.1111/j.1365-2125.1996.tb00017.x
17. Sambol, N.C., Chiang, J., O’Conner, M., Liu, C.Y., Lin, E.T., Goodman, A.M., Benet, L.Z., Karam, J.H. Pharmacokinetics and pharmacodynamics of metformin in healthy subjects and patients with noninsulin-dependent diabetes mellitus. J Clin Pharmacol, 36(11). doi: 10.1177/009127009603601105
18. Song, I.S., Shin, H.J., Shim, E.J., Jung, I.S., Kim, W.Y., Shon, J.H., Shin, J.G. (2008). Genetic Variants of the Organic Cation Transporter 2 Influence the Disposition of Metformin. Clin Pharmacol Ther, 84(5). doi: 10.1038/clpt.2008.61
